# Autologous immune enhancement therapy against an advanced epithelioid sarcoma: A case report

**DOI:** 10.3892/ol.2013.1247

**Published:** 2013-03-12

**Authors:** KANANATHAN RATNAVELU, BASKAR SUBRAMANI, CHITHRA RAMANATHAN PULLAI, KOHILA KRISHNAN, SHEELA DEVI SUGADAN, MANJUNATH SADANANDA RAO, ABHI VEERAKUMARASIVAM, XUEWEN DENG, TERUNUMA HIROSHI

**Affiliations:** 1 Nilai Cancer Institute (NCI) Hospital, Nilai 71800;; 2 Nichi-Asia Life Science Sdn. Bhd., Petaling Jaya 47810, Malaysia;; 3 Acharya Nagarjuna University, Andhra Pradesh 522510, India;; 4 Nichi-In Centre for Regenerative Medicine, Chennai 600026, India;; 5 Medical Genetics Laboratory, Faculty of Medicine and Health Sciences, Universiti Putra Malaysia, Serdang 43400, Selangor Darul Ehsan;; 6 UPM-MAKNA Cancer Research Laboratory, Institute of Bioscience, Universiti Putra Malaysia, Serdang, 43400, Selangor Darul Ehsan;; 7 Perdana University Graduate School of Medicine, Perdana University, Serdang 43400, Selangor Darul Ehsan, Malaysia;; 8 Biotherapy Institute of Japan, Tokyo 135-0051, Japan

**Keywords:** autologous immune enhancement therapy, natural killer cells, T cells, advanced epithelioid sarcoma, adverse reactions

## Abstract

Rare types of cancer are often not effectively treated by approaches such as chemotherapy and radio-therapy, although their side-effects persist. Immunotherapy has been gaining attention worldwide with growing examples of its anticancer activity demonstrated *in vivo*. This case report describes a 35-year-old male who suffered from advanced epithelioid sarcoma and underwent 18 cycles of chemotherapy without any significant response, who suffered adverse effects that caused lung collapse. A notable response was observed following the administration of autologous immune enhancement therapy (AIET), which involves a process of isolation, activation and expansion of natural killer (NK) and T cells, which were obtained from the patient’s own (autologous) peripheral blood. With the present data and the response of the patient to AIET, it may be proposed that AIET is beneficial for patients suffering from advanced epithelioid sarcoma without producing adverse effects.

## Introduction

Soft tissue sarcomas are a common type of sarcoma and account for >3,560 mortalities each year. In 2007, the incidence of soft tisue sarcomas in the United States was estimated to be 9,220 (1). However, the incidence of this heterogeneous group of mesenchymal extraskeletal malignancies, which usually occur in the extremities, trunk, retroperitoneum or head and neck, is relatively low (2–4). It is believed that the dose of radiation therapy used to treat cancer exerts a major effect on the incidence (5,6), along with other risk factors, including exposure to certain chemicals, herbicides, such as phenoxyacetic acids, and wood preservatives containing chlorophenols (7,8). The median survival rate depends on the histology subtype (3) and age at onset of the disease and is ∼1 year, with only 10% of cases surviving for up to 5 years (2). The standard treatment modalities are reported to have a minimal impact on the prognosis of the patients (2,9,10). Surgery supplemented with radiotherapy is feasible and reliable for localized diseases (11), but patients have a 50% chance of developing metastases (12). In these instancs, metastasectomy is usually attempted. In cases of inoperable recurrent disease, palliative treatment is administered, while for the metastatic form of the disease, systemic chemotherapy is usually prescribed to control disease progression and thus improve the survival rate of the patients (13–15). In order to improve the current treatment modalities, novel approaches to prevent or treat the disease are required urgently. It has been shown that the NK cells of sarcoma patients are as potent as the NK cells of healthy controls in the lysing of the tumor cells (16,17). There is an increasing amount of evidence available concerning the involvement of the immune system and how it may be manipulated in various ways to recognize and kill tumors. The effector activity of NK and T cells in reacting against the cancer antigens has been demonstrated (18), with favorable responses being observed in various tumors (19). In concordance with previous results showing the positive response of sarcomas to immunotherapy (20,21), the present study reports our experiences using *in vitro* cultured autologous NK and T cells to treat a 35-year-old male diagnosed with sarcoma in 2010.

## Case report

A 35-year-old Chinese male patient with a 1-year history of left arm swelling from unknown causes was investigated in February 2010. Written informed patient consent was obtained from the patient. Histological investigation on multiple biopsies revealed tumor tissue composed of nodular proliferation that was predominantly eosinophilic, with a few spindle cells and large areas of central necrosis and hemorrhage. The epithelial cells exhibited abundant eosinophilic cytoplasm with large vesicular nuclei and prominent cell nuclei. No vascular invasion was observed and the patient had a multifocal disease that extended along the span of the left arm in addition to the disease in the left axillaries, left clavicular and right upper planar region. Immunohistochemical analysis revealed positivity for cytokeratin (CK), EMA, vimentin and CD34, but was negative for S100, desmin, smooth muscle actin (SMA), melanin, CD68 and HMB45. The results were consistent between the left forearm and left arm epithelioid sarcoma. The patient sought advice from the sarcoma team at the Peter MacCallum Cancer Institute (East Melbourne, Australia), who advised that the patient undergo neoadjuvant chemotherapy with ifosfamide and Adriamycin. After 6 cycles, positron emission tomography scan images showed a partial response in the soft tissue density of the left upper extremity and axilla. Computed tomography scanning showed a reduction in the size of the planar base mass. After much deliberation, the patient opted to undergo autologous immune enhancement therapy (AIET).

As a therapeutic alternative, the patient received 7 infusions of AIET. The patient initially underwent an induction phase, receiving 6 harvestings of peripheral blood every 2 weeks. After a 5 month follow-up, the patient volunteered for consecutive AIET infusions. Peripheral blood (60 ml) was collected from the patient each time and peripheral blood mononuclear cells (PBMCs) were isolated from the blood as per the protocol described by Terunuma *et al*(22). Isolated PBMCs were seeded onto anti-CD3 and anti-CD16-coated flasks and incubated overnight at 39°C with 5% CO_2_. The cultures were then incubated at 37°C and maintained for 14–16 days with routine media nourishment using the patient’s own plasma separated from the peripheral blood. Interleukin-2 was added as a supplement during the NK and T cell seeding. After an optimal expansion to the desired cell population, cells were harvested by centrifugation and washed three times using phosphate-buffered saline. The retrieved cell number was measured using the Trypan blue dye exclusion test and the cells were then re-suspended in 100–200 ml of normal saline with human albumin for intravenous infusion.

## Discussion

Using the adopted method for the expansion of NK and T cells, it was determined that the average expansion of the cell population, from initial cell counts of 3.3×10^6^ NK cells and 19.3×10^6^ T cells, to final cell counts of 18.8×10^8^ and 17.6×10^8^ cells, respectively, was possible without using allogenic cells as a feeder layer to ensure the safety of the cells being infused. Thus, cell number expansions of >100-fold was achieved using the present method. Throughout the whole process, the rate of expansion was monitored and the cell density was manipulated according to the *in vitro* culture conditions. Table I shows the initial and final NK and T cell counts.

**Figure 1:**
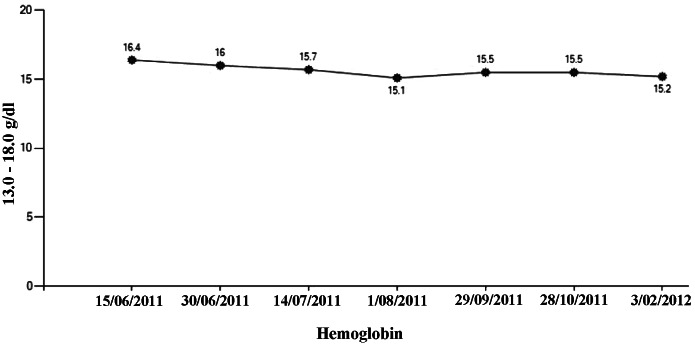
Stable hemoglobin level during the course of AIET from June 2011 to February 2012. AIET, autologous immune enhancement therapy.

It was noticeable in the expanded cell number, the large anticancer immune cell population (NK and T cells) in the cell suspension reacted actively against the tumor cells *in vivo*. The patient responded well to AIET and clear skin improvement was observed following each infusion. The patient’s chest radiography remained stable, therefore the tumor did not metastisize to the lungs. Significantly, the administered *in vitro* expanded cells did not exhibit any adverse reactions in the patient, as has been shown in numerous other studies (23). The patient opted to undergo a 3-month break and during the tail-end of this period, worsening of the skin lesion was observed. The patient decided to restart the therapy and after one infusion, and marked improvement was noted. Although the lesions persist, the fact that the patient has surpassed the anticipated survival duration is encouraging. However, certain secondary immune responses, such as wound healing, were observed. After three infusions of AIET, one of the largest tumor areas on the forearm (4×2 cm) stopped bleeding and growth of new tissue was visible, along with the recovery of smaller wounds, and during the course of the AIET, the hemoglobin level, which is believed to decrease depending on the disease severity and progression, remained stable. Fig. 1 shows the analysis of the results concerning hemoglobin levels between June 2011 and February 2012. The patient’s overall survival is at present 25 months and the patient has not received chemotherapy since August 2010. These promising results obtained with the current therapeutic approach may be an effective immunotherapeutic strategy for managing difficult tumors, such as soft tissue sarcomas (24), which were the first tumors studied for antitumor immunity (25).

In conclusion, the present case study demonstrates the efficacy of immunotherapy for a rare type of cancer that otherwise had no options for therapeutic approaches that may provide a favorable outcome in the patient. *In vitro* expanded NK and T cells were are able to respond via their anticancer activity under *in vivo* conditions without producing adverse reactions in the patient. With repeated administration of AIET, the prognosis of the patient improved as well as thei quality of life of the patient. The patient acheived stable disease without any side-effects. Therefore, the present study also demonstrates the safety and efficacy of AIET, although a larger study is required for more extensive analysis.

## Figures and Tables

**Figure 2:**
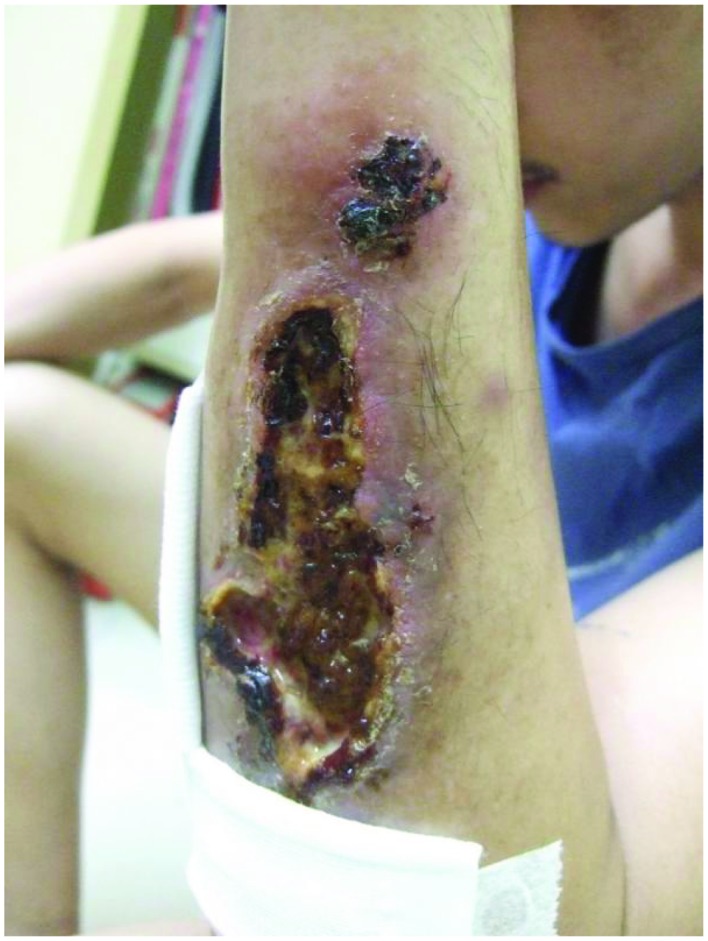

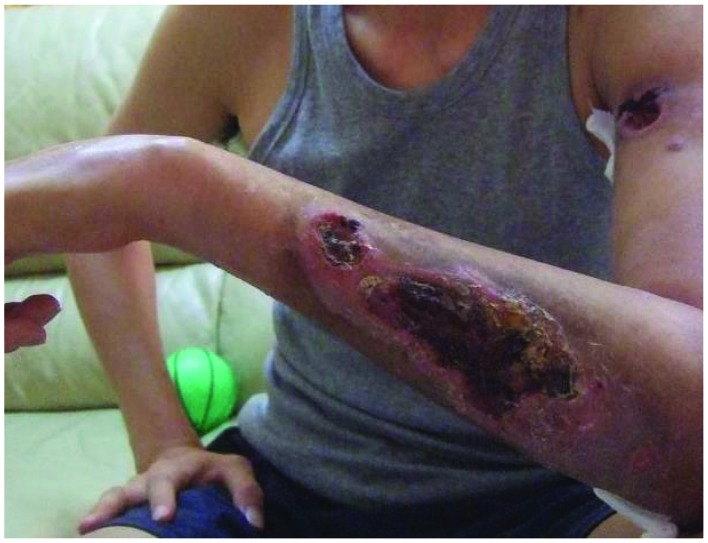

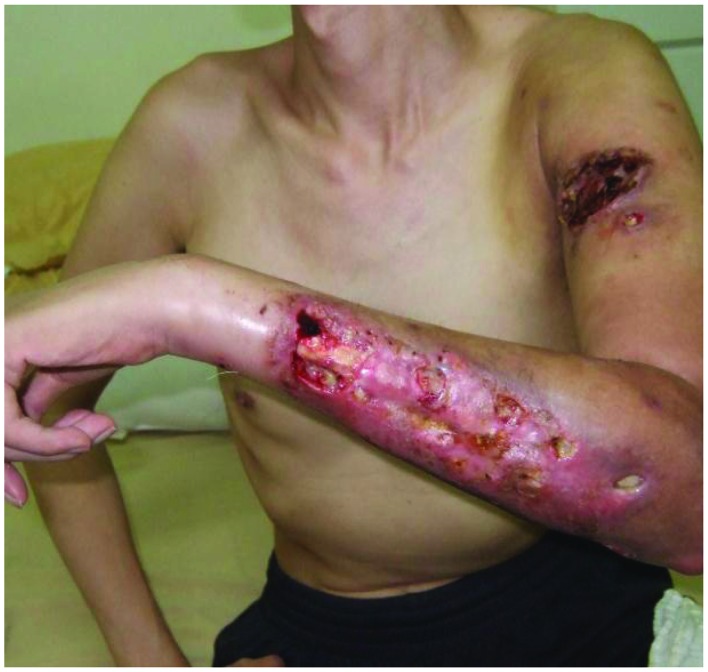
(A) Before administration of AIET, necrotic tissues with severe disease progression were observed. (B) After 3 infusions of AIET, clear healing of the wound was visible. (C) After 6 AIET infusions, significant healing of the wound was observed. AIET, autologous immune enhancement therapy.

**Table I:** Initial and final cell number of NK and T cells.

No. of infusions	Cell number (million)			
	Initial culture		Final culture	
	NK cells	T cells	NK cells	T cells
1	4.6	25.8	1,584	1,818.2
2	0.8	5.2	1,496	1,496
3	1.3	10.0	560	2,132
4	4.2	24.2	3,070	644
5	5.8	33.7	4,721	644
6	3.1	19.5	1,224	1,336
7	3.1	16.7	1,917	4,230

NK, natural killer.
